# Supplementation of Morin Restores the Altered Bone Histomorphometry in Hyperglycemic Rodents via Regulation of Insulin/IGF-1 Signaling

**DOI:** 10.3390/nu13072365

**Published:** 2021-07-10

**Authors:** Hatem M. Abuohashish, Abdullah F. AlAsmari, Mohamed Mohany, Mohammed M. Ahmed, Salim S. Al-Rejaie

**Affiliations:** 1 Department of Biomedical Dental Sciences, College of Dentistry, Imam Abdulrahman Bin Faisal University, Dammam 31441, Saudi Arabia; habuohashish@iau.edu.sa; 2 Department of Pharmacology and Toxicology, College of Pharmacy, King Saud University, Riyadh 11451, Saudi Arabia; afalasmari@ksu.edu.sa (A.F.A.); mmohany@ksu.edu.sa (M.M.); mahmedd@ksu.edu.sa (M.M.A.)

**Keywords:** diabetes, morin, bone, IGF-1, insulin, oxidative stress

## Abstract

Pathological mechanisms underlining diabetic bone defects include oxidative damage and insulin/IGF-1 imbalance. Morin is a bioflavonoid with antioxidant and anti-diabetic effects. This study evaluates morin’s protective effects against altered bone histomorphometry in diabetic rats through assessing insulin/IGF-1 pathway as a potential mechanism. Diabetic animals were administered two morin doses (15 and 30 mg/kg) for 5 weeks. Different serum hepatic and renal functions tests were assessed. Bone density and histomorphometry in cortical and trabecular tissues were evaluated histologically. The expressions of insulin, c-peptide and IGF-1 were estimated. In addition, the enzymatic activities of the major antioxidant enzymes were determined. Diabetic-associated alterations in serum glucose, aminotransferases, urea and creatinine were attenuated by morin. Diabetic bone cortical and trabecular histomorphometry were impaired with increased fibrosis, osteoclastic functions, osteoid formation and reduced mineralization, which was reversed by morin; particularly the 30 mg/kg dose. Insulin/IGF-1 levels were diminished in diabetic animals, while morin treatment enhanced their levels significantly. Diabetes also triggered systemic oxidative stress noticeably. The higher dose (30 mg/kg) of morin corrected the endogenous antioxidant enzymatic activities in diabetic rats. Findings indicate the potential value of morin supplementation against hyperglycemia-induced skeletal impairments. Activation of insulin/IGF-1 signaling could be the underlining mechanism behind these effects.

## 1. Introduction

Diabetes mellitus is a well-known metabolic disease affecting millions of people around the world. The metabolic disturbance during diabetes leads to multiple deleterious effects on different body organs. One of the major diabetic complications is skeletal impairments and reduction in bone mass. The incidence of diabetic-associated bone loss is escalating in relation to the global spread of the metabolic disease [1]. Moreover, several clinical and experimental studies have indicated that diabetes may increase the risk of fractures [1]. On the cellular level, diabetic-induced hyperglycemia may impair bone metabolism and mineralization leading to altered bone remodeling and imbalanced osteoclastogenesis and osteoblastogenesis ratio [2]. Several pathological mechanisms were suggested to explain bone deformities associated with diabetes. Involvement of inflammatory cytokines, generation of advanced glycated end products, development of reactive oxygen species (ROS) and oxidative stress are examples of these caustic pathways [2]. Other studies suggested that discrepancy in insulin like growth factor (IGF-1) expression might contribute directly to bone health and quality in diabetic patients [3]. IGF-1 axis was found to improve bone growth and minerals content in diabetic children [4]. Low levels of plasma IGF-1 are correlated positively with vertebral fractures in type 2 diabetic postmenopausal female patients [5]. Furthermore, studies conducted on experimental animals provided remarkable evidences about the crucial role of IGF-1 in skeletal acquisition and strength. In one study, the promoted production of IGF-1 improved osteoblastic proliferation and differentiation [6]. In another study, the osteo-protective effects of vitamin D were associated with enhanced IGF-1 content in diabetic rats [7]. Reports showed that medications anti-diabetic activities could alleviate bone deformities during diabetes through regulation of insulin, glucose and IGF-1 levels [8].

The anti-diabetic properties of several flavonoids were suggested in multiple studies. Morin is a naturally occurring flavonoid with various pharmacological actions [9]. The anti-diabetic, hypoglycemic and insulin mimicking actions of morin are well-documented in numerous studies [10,11,12]. Interestingly, studies have shown that morin could enhance bone health and metabolism and reestablish skeletal functions and remodeling during several pathological conditions. In the study of Wang et al., morin palliated bone loss associated with glucocorticoid therapy through modulation mitogen activated protein kinase signaling [13], while down-regulated receptor activator of nuclear factor kappa-Β ligand (RANKL) expression, a well-known regulator for osteoclastic development and activity, in the study of Sultana et al. using a model of osteoarthritis [14]. Notably, one of our previous studies demonstrated morin’s ability to reduce inflammatory cytokines and to enhance bone microarchitecture in diabetic animals [15]. However, morin’s effects on bone histomorphometry and the mechanistic pathway behind this protection were not assessed. Here, we conducted a histomorphometric analysis of trabecular and cortical bone in diabetic animals. In addition, the involvement of insulin/IGF-1 signaling as an underlining pathway of morin effects on type-1 diabetic skeletal tissues was documented in the present study.

## 2. Materials and Methods

Animals and diabetes model: This study employed twenty-four male Wister albino rats (8 to 10 weeks old), which approximately weigh 260–280 g. Animals were housed in the Experimental Animal Care Center, College of Pharmacy, King Saud University. The uses of experimental animals as well as all experimental procedures were ethically approved by the Research Ethics Committee (REC) at the King Saud University based on the recommendation of the Research Ethics Sub-Committee (Ethics Reference No: KSU-SE-20-78). In addition, the Guide for the Care and Use of Laboratory Animals, 8th edition (NIH Publications 2011) was followed in the present study. Sample size was calculated using online free software (www.calculator.net, accessed on 2 November 2020) based on the results of our previous experiment [15], assuming a 95% confidence level, 5% margin of error and (50%) population proportion. The experimental chemical model utilized in the present study to induce type-1 diabetes was injection of streptozotocin intraperitoneally in a dose of 60 mg/kg body weight (Sigma Aldrich, St. Louis, MO, USA). Streptozotocin was freshly prepared in 0.1 mol/L citrate buffered solution (pH 4.5). Six control animals were injected with equal volume of streptozotocin vehicle. Diabetes was confirmed in streptozotocin injected animals after 4 days by estimating the fasting glucose levels in blood samples obtained from each animal tail vein. Animals which showed fasting blood glucose levels more than 300 mg/dL were included as diabetic animals.

Study design: All animals were allocated into four groups by adding 6 animals in each group (*n* = 6). The first group (Group-I) included normal non-diabetic animals with no treatment. The second group (Group-II) included diabetic animals with no treatment. The third and fourth groups (Group-III and Group-IV) included diabetic animals with morin oral treatment by gavage in two doses (15 and 30 mg/kg/day respectively). Doses of morin were selected according to previous studies [15]. Morin treatment started one week after diabetes induction in the animals. The daily treatment was continued for five consecutive weeks, where the animal’s health and behavior were monitored carefully. Animals had free access to food and water *ad libitum*. All animals had standard rat chow diet consists mainly of 48% carbohydrates, 21% protein, 4% fat, 6% fibers, 2% minerals, 13% moisture and 6% ash (total energy 306 kcal/100 g). Animals were then sacrificed under the light general anesthesia by ketamine. Serum samples were collected after centrifugation of blood samples at 4000 RPM for 15 min. The right and left femurs bones of each animal were dissected and preserved for the histological analysis.

Bone density determination: The left femoral bone density of each animal was measured volumetrically by Archimedes’ principle. In brief, each bone was degassed in deionized water. Bone samples were weighted before and after removal from the water [16].

Biochemical analysis: Serum levels of glycated hemoglobin (HbA1c), blood glucose level (BGL), alkaline phosphatase (ALP), alanine aminotransferase (ALT), aspartate aminotransferase (AST), blood urea nitrogen (BUN) and creatinine (Cr) were determined using the commercially available kits according to manufacture instructions (RANDOX Laboratories Ltd., Moorgate, UK).

Histological examinations: The trabecular and cortical femur bone histomorphometry were determined as described by Tamagaki et al. [17] with modifications. In brief, the right femur of each animal was trimmed of muscle and other surrounding tissues. Bones samples were fixed in 10% neutral buffered formalin. Next, the undecalcified bone samples were embedded in plastic resin. The blocks were sectioned using a microtome (American Optical Rotary Microtome, Middleton, WI, USA) into 5 µm-thick sections and stained with hematoxylin and eosin. Osteoclasts were identified by TRAP staining (Sigma 387-A, St. Louis, MO, USA). TRAP staining was used to identify osteoblasts/osteoclasts and then osteoblasts surface inside the bone matrix was considered as osteoid surface. The stained sections were mounted and observed for histopathological changes in a blinded fashion manner using a Leica DM5500 B microscope (Leica Biosystems Melbourne Pty Ltd., Melbourne, VIC, Australia). Parameters for trabecular and cortical histomorphometry were defined and nominated in accordance with American Society for Bone and Mineral Research (ASBMR) nomenclature.

Assessment of insulin/IGF-1 pathway: The expressions of insulin, c-peptide and IGF-1 in serum were determined quantitatively using enzyme-linked immunosorbent assay (ELISA) technique following instruction provided by the kits (USCN LIFE, Wuhan EIAab Science Co., Ltd., Wuhan, China).

Antioxidant enzymes assay: The enzymatic activities of the antioxidant enzymes including superoxide dismutase (SOD), catalase (CAT), glutathione peroxidase (GPx) and glutathione S transferase (GST) were evaluated in serum by commercially available kits (Cyman Chemical Company, Ann Arbor, MI, USA).

Statistical analysis: The quantitative values of the measured parameters were expressed in each group as mean ± standard error of the mean (S.E.). The statistical analysis in this study was conducted via one way analysis of variance (ANOVA) followed by Tukey post hoc test. Group-I was compared to diabetic groups, while Group-II was compared to Group-III and Group-IV. GraphPad Prism 5 was used as analyzing software (GraphPad Software, Inc., La Jolla, CA, USA). Pearson correlation was employed to test the association between different numerical variables. Heat maps and pairwise analysis was performed using Heatmapper (Wishart Research Group, University of Alberta, Ottawa, ON, Canada) [18]. The significances were considered when *p* < 0.05.

## 3. Results

Experimentally induced type-1 diabetes significantly lowered the final body weight (*p* < 0.001), bone weight (*p* < 0.01) and bone density (*p* < 0.01) of diabetic animals, while bone weight/100 g final body weight ration were altered but not significantly, as compared to control animals in group-I. Treatment of the diabetic animals with morin significantly improved the altered final body weight (*p* < 0.01), bone weight (*p* < 0.05) and density (*p* < 0.05) in diabetic animals as compared to group-II (Table 1). Serum biochemistry in normal and diabetic animals revealed significantly increased serum values of HbA1c (*p* < 0.05), BGL (*p* < 0.001) and ALP (*p* < 0.05) as compared to group-I. Diabetic animals treated with morin (15 mg/kg) showed significantly higher BGL (*p* < 0.05) as compared to control animals in group-I. The higher dose of morin (30 mg/kg) demonstrated more prominent restorative effects against the diabetic-altered serum levels of BGL (*p* < 0.05) as compared to diabetic animals in group-II (Table 2).

There were a considerable altered histomorphometry of the micro-structure of the femoral bones in diabetic animals, which was manifested by increased cortical osteoclast surface/bone surface (*p* < 0.01), porosity area/cortical bone area (*p* < 0.05), osteoid volume/cortical bone area (*p* < 0.01) and fibrosis tissue volume/cortical bone area (*p* < 0.001), while mineralizing surface/osteoid surface (*p* < 0.001) was decreased, in diabetic group-II as compared to non-diabetic animals in group-I. Diabetic animals treated with morin in the 30 mg/kg dose had a markedly enhanced osteoclast surface/bone surface (*p* < 0.05) and fibrosis tissue volume/cortical bone area (*p* < 0.05) values as compared to diabetic untreated group-II (Table 3). Similarly, the trabecular histomorphometry of the femoral bones micro-structure was impaired in diabetic animals. Values of osteoclast surface/bone surface, osteoid volume/bone volume and fibrosis tissue volume/tissue volume were significantly (*p* < 0.01, *p* < 0.001 and *p* < 0.01, respectively) increased, while trabecular thickness (*p* < 0.001), trabecular number (*p* < 0.01) and mineralizing surface/osteoid surface (*p* < 0.01) values were significantly reduced, in diabetic untreated animals as compared with group-I. Diabetic animals treated with 30 mg/kg morin for 5 weeks showed corrected trabecular histomorphometry as represented by significant decreased osteoclast surface/bone surface (*p* < 0.05) and fibrosis tissue volume/tissue volume (*p* < 0.05), while increased trabecular thickness (*p* < 0.05) and trabecular number (*p* < 0.05), in diabetic animals as compare to untreated diabetic group-II (Table 4).

**Figure 1:**
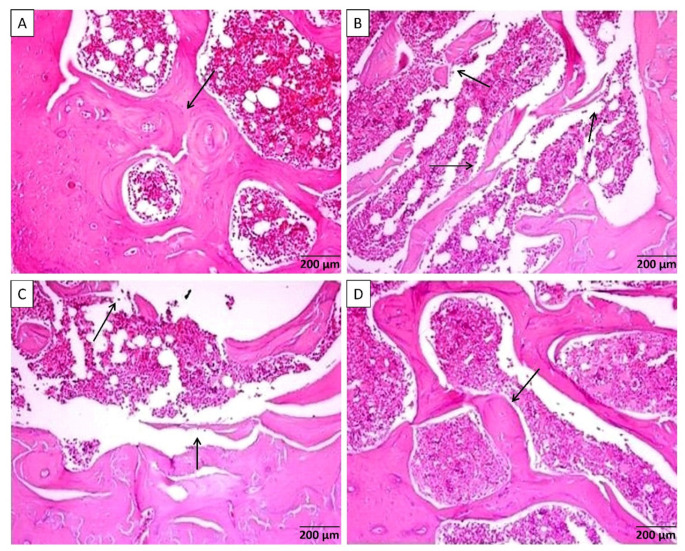
(**A**) Rat femur sections from control group-I showed benign looking bone cortex and trabeculae [arrow] with normal mineralization separated by bone marrow element. (**B**) Sections from diabetic group-II with osteoporotic changes showing thickening of bone cortex associated with separated bone trabeculae [arrows] containing bone marrow element. (**C**) Rat femurs from group-III revealed fewer thinning of bone trabeculae [arrows] and some degree of separation seen containing bone marrow elements. (**D**) Microscopical description of group-IV femurs revealed well-formed bone trabeculae [arrow] with benign looking thickness and mineralization with joined trabeculae separated by bone marrow element.

Histological screening of femurs bones from group-I showed normally looking tissues without cortical or trabecular thinning, separations, or demineralization. However, sections from diabetic group-II demonstrated osteoporotic changes including thinning of bone cortex along with separation in bone trabeculae indicating moderated osteoporotic changes. Diabetic group-III treated with morin (15 mg/kg) revealed a slight thinning in bone trabeculae and some degree of separation indicating mild osteoporotic changes. Diabetic group-IV treated with morin (30 mg/kg) demonstrated a well formed benign looking joined bone trabeculae and cortex with a minimal degree of bone thinning and demineralization (Figure 1).

**Figure 2:**
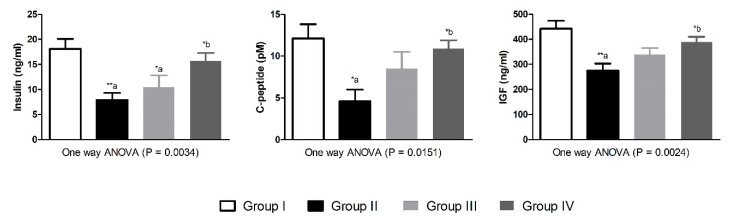
Effects of morin on insulin/IGF-1 levels in normal and diabetic Wistar rats. Data were expressed as Mean ± S.E. (*n* = 6). Statistical analysis was conducted by one-way ANOVA followed by Tukey post hoc test. The significances were considered as follow (*) *p* < 0.05 and (**) *p* < 0.01. Group-I was compared to diabetic groups (a), while Group-II was compared to Group-III and Group-IV (b).

**Figure 3:**
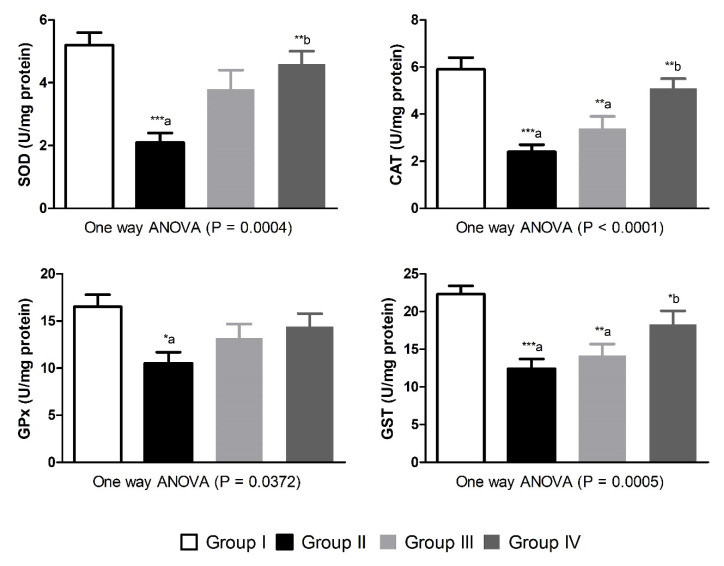
Effects of morin on antioxidant enzymes activities in normal and diabetic Wistar rats. Data were expressed as Mean ± S.E. (*n* = 6). Statistical analysis was conducted by one-way ANOVA followed by Tukey post hoc test. The significances were considered as follow (*) *p* < 0.05, (**) *p* < 0.01 and (***) *p* < 0.001. Group-I was compared to diabetic groups (a), while Group-II was compared to Group-III and Group-IV (b).

Induction of type-1 diabetes in the experimental animals resulted in significant reduction in serum values of insulin and c-peptide (*p* < 0.01 and *p* < 0.05, respectively) as compared to control animals. Moreover, the levels of IGF-1 were significantly (*p* < 0.01) lower in the diabetic group-II compared to non-diabetic animals. Treatment of the diabetic animals with morin for 5 weeks, particularly the 30 mg/kg dose, markedly (*p* < 0.05) corrected the altered values of insulin, c-peptide and IGF-1 as compared to diabetic animals in group-II (Figure 2). The antioxidant enzymatic activities of SOD, CAT, GPx and GST were significantly (*p* < 0.001, *p* < 0.001, *p* < 0.05 and *p* < 0.001, respectively) decreased in diabetic untreated animals as compared with group-I. Morin treatment in 15 mg/kg dose could not increase the antioxidant enzymatic activities significantly. However, the 30 mg/kg dose of morin significantly restored the antioxidant enzymatic activities of SOD, CAT and GST (*p* < 0.01, *p* < 0.01 and *p* < 0.05, respectively) as compared with group-II diabetic animals (Figure 3).

**Figure 4:**
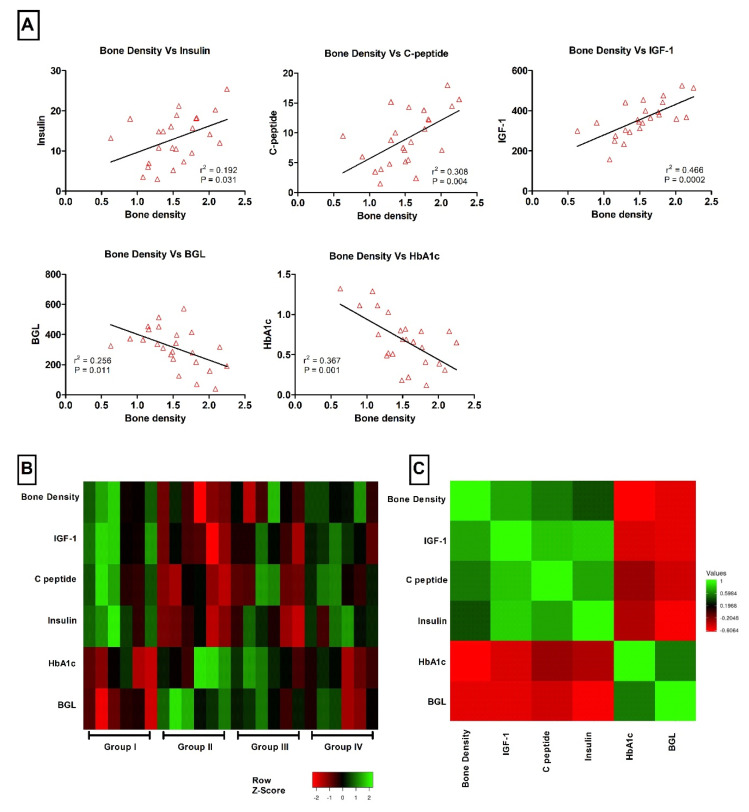
Correlation analysis between bone density and multiple variables including insulin, C peptide, IGF-1, BGL and HbA1c. (**A**) Correlation between bone density and insulin, C peptide, IGF-1, BGL and HbA1c, respectively. *p* values and r2 are expressed on each figure. (**B**) Heat map showing the distribution of bone density and insulin, C peptide, IGF-1, BGL and HbA1c as Z score between groups. (**C**) Heat map showing the pairwise Pearson correlation analysis expressed as r values.

Figure 4 showed the correlation between bone density and other different variables including insulin, C peptide, IGF-1, BGL and HbA1c. Results revealed positive significant correlation between bone density and insulin, C peptide and IGF-1 (*p* < 0.05, *p* < 0.01 and *p* < 0.001, respectively). A negative correlation between bone density and BGL and HbA1c was reported (*p* < 0.05 and *p* < 0.01, respectively). Values of r2 are expressed on the Figure 4A. Distribution of bone density, insulin, C peptide, IGF-1, BGL and HbA1c values among different groups as Z score is expressed in Figure 4B. Pairwise analysis, shown in Figure 4C, demonstrating positive Pearson correlation values between bone density and insulin, C peptide and IGF-1, while negative Pearson correlation values between bone density and BGL and HbA1c.

## 4. Discussion

One important metabolic complication during diabetes is bone loss, which might lead to osteopenia. Several studies reported skeletal abnormalities a long with altered bone metabolism as a chronic complication in type 1 and 2 diabetes [1]. Moreover, it has been suggested that chronic alterations in glucose and IGF-1 levels are risk factors for bone fragility during diabetes, which may lead to bone remodeling and impaired formation, which could be alleviated by several anti-diabetic medications [6,7,8]. The present study documented the protective effects of morin systemic treatment against skeletal structural abnormalities in type-1 diabetic rats. Morin treatment showed marked restorative effects against diabetic-induced oxidative stress and impaired bone histomorphometry. This study also suggests that the reported bone protective properties of morin are mediated through the insulin/IGF-1 signaling pathway.

In this study, we utilized the streptozotocin induced type-1 diabetes model in experimental animals. When streptozotocin reaches to the circulation, it is selectively up taken by beta cells in the pancreas by the action of glucose transporter type 2. Inside the beta cells, streptozotocin cases DNA alkylation, which results in loss of cellular function and prompt decrease in insulin levels leading to hyperglycemia [19]. Hyperglycemia is a major risk factor for cellular damage and production of free radicals. Generation of ROS during hyperglycemia may exceed the endogenous antioxidant capacity leading to multiple oxidative damages in several organs. Therefore, it could be anticipated that diabetic-induced oxidative stress plays a vital pathological role in diabetic complications. We reported that the antioxidant enzymatic activities of SOD, CAT, GPx and GST were diminished in diabetic animals. The deleterious effects of ROS and other free radicals are endogenously detoxified by multiple cellular defense mechanisms including antioxidant enzymes, which restore the cellular functions. SOD is an antioxidant enzyme that transforms oxygen radicals like O_2_- to the less harmful species like H_2_O_2_, which is then hydrolyzed to water and oxygen by the action of CAT (another antioxidant enzyme). On the other hand, antioxidant enzymes like GPx and GST mediate the transformation of two reduced glutathione (GSH) molecules to GSSG. This reaction will produce two water molecules after reduction of one H_2_O_2_ molecule. Six weeks of hyperglycemia and the resulted oxidative stress were enough to markedly disturb bone density and morphometry. These results are in harmony with previous studies, where alterations in bone microstructure were reported in rats with a period of 6 weeks or less of type-1 diabetes using similar STZ model [20,21]. In addition, the present BMD values are higher than previous reported values [15] due to different techniques (micro-CT vs. *Archimedes’ principle*) and measurement areas (femoral heads vs. whole femoral bone).

Morin is a bio-active natural compound with multiple pharmacological effects. Interestingly, morin showed antidiabetic effects in several studies [12]. In the current study, morin treatment slightly, but not significantly, corrected the alter levels of liver and kidney function biomarkers including ALT, AST, ALP, BUN and Cr, which might suggest the protective role of morin against the metabolic changes associated with diabetes. In addition, these effects are in accordance with multiple studies, where the protective properties of morin were deemed to be due to antioxidant and free radical scavenger properties [22]. We also reported the antioxidant value of morin in the current study. Morin treatment obviously improved the antioxidant enzymatic activities of SOD, CAT and GST. The antioxidant effects of morin might be mediated via the presence of hydroxyl groups in its chemical structure. Morin and other bioflavonoids have multiple hydroxyl groups in their aromatic and c-pyrone rings. These groups are able to scavenge free radicals, which reduces their oxidative damaging effects [22], particularly on the skeletal system. This might suggest the therapeutic value of morin against diabetic-induced bone impairments.

In the current study, morin showed protective effects against diabetic-induced alterations in micro-architecture of bone tissues. Morin markedly enhanced the cortical and trabecular bone histomorphometry in diabetic animals and prevented the fibrotic effects of hyperglycemia in cortical and trabecular bone. Studies reported the osteo-protective effects of morin in different animal model including glucocorticoid-induced osteoporosis [13]. Morin also down-regulated the osteoclastogenesis-regulating factors in an animal model of osteoarthritis [14]. Interestingly, we previously reported bone restorative effects of morin in diabetic osteopenia, which was confirmed by micro-CT analysis. The anti-inflammatory effects of morin were suggested as mechanistic function in this study [15]. However, the novelty aspect in this study is that the determination of morin effects on bone histomorphometry along with the suggestion of insulin/IGF-1 as another protective pathway by which morin might protect skeletal tissues from the deleterious effects of hyperglycemia.

Studies have shown that IGF-1 has two types of receptors. The first type is a specific IGF-1R, while the second type is a hybrid IGF-1/insulin receptor. IGF-1 binds selectively to both types of receptor leading to intracellular signaling. IGF-1 signaling, together with insulin/insulin receptor signaling, mediates cellular proliferation and survival in multiple pathological conditions including hyperglycemia [23]. These favorable physiological effects were also reported in the skeletal system. Several studies documented the vital role of IGF-1 in organic skeletal matrix. IGF-1 was found to enhance the differentiation osteoblasts and regulate bone turn over. IGF-1 boosts bone longitudinal growth and mas especially during childhood [24]. In addition, studies have indicated that low levels of serum IGF-1 are correlated with reduced bone density and bone fractures [25]. Here, we reported that the serum levels of insulin, c-peptide and IGF-1 were lower in diabetic untreated animals that control non-diabetic ones, which suggest the enrolment of insulin/IGF-1 in diabetic osteopenia.

The osteo-protective effects of morin after 5 weeks of treatment to diabetic animals were associated with enhanced insulin, c-peptide and IGF-1 serum levels, which is accordance with previous finding from other studies. In the study of Lin et al., the cultured pancreatic cells had marked insulin secretion when treated by morin, through imidazoline I-3 receptor dependent pathway, indicating its usefulness in treating diabetic related disorders [12]. Morin enhanced insulin secretion in diabetic rats in another in vivo study [10]. Moreover, morin was suggested as a non-competitive inhibitor for protein tyrosine phosphatase type 1B, leading to insulin receptor sensitization and activation [11]. Notably, we previously reported that morin treatment may reduce the levels of IGF-1 in the brain tissues of diabetic animals, which was associated with attenuated inflammation and oxidative stress [26]. Therefore, it could be suggested that the skeletal protective effects of morin against diabetic bone loss are mediated through its ability to enhance insulin secretion and receptor sensitivity beside its pivotal role in potentiating IGF-1 production.

This study has two major limitations. The first one is that we did not investigate the morin effects in female animals, which omitted the hormonal role in mediating its osteo-protective effects. The second limitation is that the concentrations of minerals including calcium and inorganic phosphorus were not measured in serum or urine samples. However, this could be compensated by the estimation of the skeletal degree of mineralization histologically in femoral bones.

## 5. Conclusions

Taken together, the biochemical and histological findings of the present study provide experimental evidence regarding the therapeutic value of morin against metabolic complications associated with diabetes. Morin reduced skeletal impairments and bone loss in diabetic animals. The underlining mechanism of these reported osteo-protective effects could be elucidated through insulin/IGF-1 signaling. These findings may highlight the clinical relevance of morin as a nutritional supplement. The estimated adult human equivalent doses [27] of morin corresponding to the selected experimental doses are 2.5 to 5 mg/kg. Accordingly, morin could be promoted clinically for testing its usefulness in treatment of diabetic related disorders.

## Figures and Tables

**Table 1:** Effects on body and bone weights and density in normal and diabetic Wistar rats.

	Abbreviation	Unit	Group-I	Group-II	Group-III	Group-IV	One Way ANOVA *p* Value
Initial Body weight	IBW	g	275 ± 4.8	286 ± 6.2	283 ± 6.1	268 ± 5.5	0.1396
Final Body weight	FBW	g	321 ± 8.9	209 ± 12 ***^,a^	233 ± 10 ***^,a^	266 ± 9 **^,a,b^	<0.0001
Bone weight	BW	g	0.59 ± 0.03	0.44 ± 0.03 **^,a^	0.48 ± 0.02	0.54 ± 0.03 *^,b^	0.0058
Bone density	BMD	g/cm^3^	1.82 ± 0.12	1.16 ± 0.13 **^,a^	1.44 ± 0.16	1.67 ± 0.09 *^,b^	0.0088
Bone weight/100 g final body weight	BW/100FBW	%	0.184 ± 0.019	0.234 ± 0.031	0.221 ± 0.017	0.218 ± 0.027	0.5202

Data were expressed as Mean ± S.E. (*n* = 6). Statistical analysis was conducted by one-way ANOVA followed by Tukey post hoc test. The significances were considered as follow (*) *p* < 0.05, (**) *p* < 0.01 and (***) *p* < 0.001. Group-I was compared to diabetic groups ^a^, while Group-II was compared to Group-III and Group-IV ^b^.

**Table 2:** Effects on serum biochemistry in normal and diabetic Wistar rats.

Biochemical Parameters	Abbreviation	Unit	Group-I	Group-II	Group-III	Group-IV	One Way ANOVA *p* Value
Glycated hemoglobin	HbA1c	%	0.41 ± 0.11	0.95 ± 0.14 *^,a^	0.82 ± 0.09	0.53 ± 0.08	0.0068
Blood glucose level	BGL	mg/dL	178 ± 42	444 ± 37 ***^,a^	343 ± 28 *^,a^	280 ± 49 *^,b^	0.0012
Alkaline Phosphatase	ALP	U/L	206 ± 47	1183 ± 388 *^,a^	813 ± 157	652 ± 169	0.0462
Alanine aminotransferase	ALT	U/L	43.35 ± 4.46	66.62 ± 11.41	47.50 ± 6.10	55.16 ± 7.28	0.1912
Aspartate Aminotransferase	AST	U/L	78.03 ± 10	108.99 ± 13	83.57 ± 11	89.50 ± 8	0.2204
Blood urea nitrogen	BUN	µm/L	66.31 ± 22	183.30 ± 51	129.85 ± 15	131.67 ± 27	0.1114
Creatinine	Cr	µm/L	64.71 ± 22	85.97 ± 6	54.58 ± 28	61.71 ± 15	0.7018

Data were expressed as Mean ± S.E. (*n* = 6). Statistical analysis was conducted by one-way ANOVA followed by Tukey post hoc test. The significances were considered as follow (*) *p* < 0.05 and (***) *p* < 0.001. Group-I was compared to diabetic groups ^a^, while Group-II was compared to Group-III and Group-IV ^b^.

**Table 3:** Quantitative analysis of femur histomorphometry (cortical bone) in normal and diabetic Wistar rats.

Histomorphometry Parameters	Abbreviation	Unit	Group-I	Group-II	Group-III	Group-IV	One Way ANOVA *p* Value
Osteoclast surface/bone surface	Oc.S/BS	%	1.9 ± 0.09	3.2 ± 0.4 **^,a^	2.8 ± 0.3	2.1 ± 0.1 *^,b^	0.0065
Porosity area/cortical bone area	Po.Ar/Ct.Ar	%	2.1 ± 0.5	4.2 ± 0.4 *^,a^	3.4 ± 0.4	2.6 ± 0.4	0.0126
Osteoid volume/cortical bone area	OV/Ct.Ar	%	1.4 ± 0.3	3.6 ± 0.5 **^,a^	2.9 ± 0.55	2 ± 0.3	0.0084
Mineralizing surface/osteoid surface	MS/BS	%	57.1 ± 6	18.9 ± 4 ***^,a^	28.6 ± 5 **^,a^	38.4 ± 6	0.0004
Fibrosis tissue volume/cortical bone area	Fb.V/Ct.Ar	%	0.02 ± 0.009	0.2 ± 0.04 ***^,a^	0.1 ± 0.03	0.08 ± 0.02 *^,b^	0.0015

Data were expressed as Mean ± S.E. (*n* = 6). Statistical analysis was conducted by one-way ANOVA followed by Tukey post hoc test. The significances were considered as follow (*) *p* < 0.05, (**) *p* < 0.01 and (***) *p* < 0.001. Group-I was compared to diabetic groups ^a^, while Group-II was compared to Group-III and Group-IV ^b^.

**Table 4:** Quantitative analysis of femur histomorphometry (trabecular bone) in normal and diabetic Wistar rats.

Histomorphometry Parameters	Abbreviation	Unit	Group-I	Group-II	Group-III	Group-IV	One Way ANOVA *p* Value
Osteoclast surface/bone surface	Oc.S/BS	(%)	5.4 ± 2.1	19.2 ± 3.1 **^,a^	12.4 ± 2.4	9.5 ± 1.6 *^,b^	0.0042
Trabecular thickness	Tb.Th	(µm)	69.4 ± 3	30.4 ± 6 ***^,a^	43.5 ± 5 **^,a^	51.2 ± 4 *^,b^	<0.0001
Trabecular number	Tb.N	(mm)	3.5 ± 0.14	2.5 ± 0.2 **^,a^	2.9 ± 0.12	3.2 ± 0.2 *^,b^	0.0032
Osteoid volume/bone volume	OV/BV	(%)	8.3 ± 1.6	37.4 ± 6.3 ***^,a^	28.5 ± 5.5 *^,a^	21.2 ± 2.9	0.0015
Mineralizing surface/osteoid surface	MS/BS	(%)	37.4 ± 4.3	17.4 ± 3.1 **^,a^	27.3 ± 4.2	32.2 ± 3.5	0.0095
Fibrosis tissue volume/tissue volume	Fb.V/TV	(%)	0.1 ± 0.003	0.35 ± 0.07 **^,a^	0.2 ± 0.04	0.12 ± 0.05 *^,b^	0.0054

Data were expressed as Mean ± S.E. (*n* = 6). Statistical analysis was conducted by one-way ANOVA followed by Tukey post hoc test. The significances were considered as follow (*) *p* < 0.05, (**) *p* < 0.01 and (***) *p* < 0.001. Group-I was compared to diabetic groups ^a^, while Group-II was compared to Group-III and Group-IV ^b^.

## Data Availability

The data presented in this study are available in the article.
